# Potential Geographic Distribution of the Novel Avian-Origin Influenza A (H7N9) Virus

**DOI:** 10.1371/journal.pone.0093390

**Published:** 2014-04-01

**Authors:** Gengping Zhu, A. Townsend Peterson

**Affiliations:** 1 Tianjin Key Laboratory of Animal and Plant Resistance, College of Life Sciences, Tianjin Normal University, Tianjin, China; 2 Biodiversity Institute, University of Kansas, Lawrence, Kansas, United States of America; University of Minnesota, United States of America

## Abstract

**Background:**

In late March 2013, a new avian-origin influenza virus emerged in eastern China. This H7N9 subtype virus has since infected 240 people and killed 60, and has awakened global concern as a potential pandemic threat. Ecological niche modeling has seen increasing applications as a useful tool in mapping geographic potential and risk of disease transmission.

**Methodology/Principals:**

We developed two datasets based on seasonal variation in Normalized Difference Vegetation Index (NDVI) from the MODIS sensor to characterize environmental dimensions of H7N9 virus. One-third of well-documented cases was used to test robustness of models calibrated based on the remaining two-thirds, and model significance was tested using partial ROC approaches. A final niche model was calibrated using all records available.

**Conclusions/Significance:**

Central-eastern China appears to represent an area of high risk for H7N9 spread, but suitable areas were distributed more spottily in the north and only along the coast in the south; highly suitable areas also were identified in western Taiwan. Areas identified as presenting high risk for H7N9 spread tend to present consistent NDVI values through the year, whereas unsuitable areas show greater seasonal variation.

## Introduction

In March and early April 2013, a new avian-origin influenza A (H7N9) virus emerged in eastern China, this H7N9 is causing disease in humans. People infected with H7N9 usually showed symptoms including fever, coughing, and severe acute respiratory illness, and the virus has now infected at least 240 and killed 60 people in China, causing global concern regarding potential pandemic threats [1]–[2]. Since the first case was reported, researchers focused on improving diagnosis, understanding location of origin, and methods of cure; however, little is known about the geographic potential of H7N9 or environmental correlates of its transmission, except Butler [3] and He and Chen [4], who presented ideas based on lessons from the previous avian influenza threat (i.e. H5N1), and Shi [5] and Fang [6], who mapped the spread potential of H7N9 using spatial regression method.

**Figure 1:**
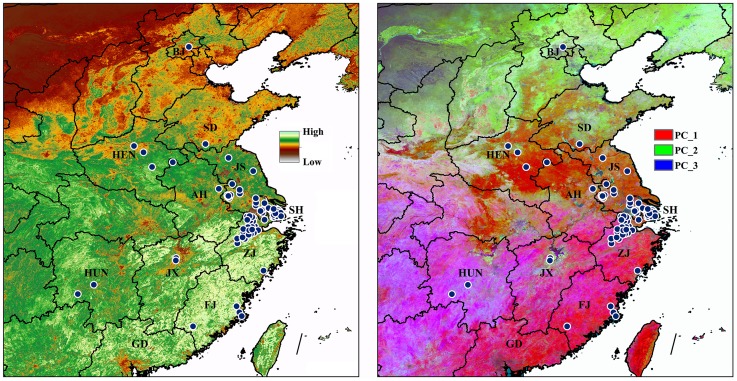
Known H7N9 virus cases across eastern China (blue dots) overlaid on the bioNDVI layers (left), and principal components analysis visualizations (right) of environmental variation across eastern China. Simplified province names were overlaid on the map (LN: Liaoning, SD: Shandong, HEN: Henan, JS: Jiangsu, AH: Anhui, HB: Hubei, SH: Shanghai, ZJ: Zhejiang, JX: Jiangxi, HUN: Hunan, FJ: Fujian, GD: Guangdong, TW: Taiwan).

The first known human H7N9 infections were reported on 31 March 2013, with two cases in Shanghai and one in the neighboring province of Anhui. By 22 April 2013, the World Health Organization (WHO) tallied 104 confirmed cases, over an expanded area including neighboring Jiangsu and Zhejiang provinces [3]. Most human H7N9 cases remain concentrated around Shanghai, but cases have been detected in Beijing in the north, Henan and Anhui provinces in the interior, and Hunan, Jiangxi, Fujian and Guangdong provinces in the south (Figure 1). The source of H7N9 human infections is unclear, but it appears to be carried by poultry, in their secretions or excretions [7]–[8].

Ecological niche modeling (ENM) offers a means by which to characterize environmental conditions suitable for species or phenomena such as disease transmission, and identify where those suitable environments are manifested in geographic space [9]. Although applications of ENM to understanding the geography and ecology of disease transmission are in early stages of exploration and development, it has been successfully applied to transmission of several diseases (e.g. [10]–[11]), including avian influenza (e.g. H5N1; [12]). This contribution aims to present a first range-wide ENM-based analysis of H7N9 in China.

## Materials and Methods

### H7N9 case occurrences

H7N9 cases have been documented in detail since the initiation of the outbreak; in particular, a real-time H7N9 reporting system was launched (http://goo.gl/maps/ZsVW8; [1]), from where we obtained 87 georeferenced occurrences for analysis; an additional 9 points were obtained from the literature or Internet (Table S1 in Supporting Information). Only the laboratory confirmed cases were used in this study. The 96 occurrence points varied in terms of spatial clumping, so we subsampled them to reduce sampling bias and effects of spatial autocorrelation, as follows. We first generated models using all available occurrence points, and measured spatial autocorrelation among model pseudoresiduals (1 – probability of occurrence generated by model) by calculating Moran's *I* at multiple distance classes using SAM v4.0 [13]; significance was determined using permutation tests. A minimum distance of 13 km was detected, after which we created a grid with cell dimensions of 0.117×0.117°, and selected the occurrence point closest to the centroid of each grid cell; this procedure reduced the number of occurrences to 77 points for final model calibration.

### Environmental data

We sought environmental data that would match the occurrence data in spatial resolution and temporal coverage, and provide a rich characterization of environmental landscapes for model calibration. The best match was multitemporal Normalized Difference Vegetation Index (NDVI) data from the MODIS (Moderate Resolution Imaging Spectroradiometer) satellite. Sixteen-day NDVI composites were obtained for January 2012 to June 2013 at a resolution of 1 km^2^ (http://modis.gsfc.nasa.gov/data/). We used two approaches by which to reduce dimensionality from the initial 34 data layers. The first was to extract the first 10 principal component axes to summarize major axes of variation among the 34 NDVI layers (97.5% of overall variation); this dataset captured major dimensions of environmental variation across eastern China. The second ‘bioNDVI’ was based on all biweekly images from January 2012 – June 2013, transformed into data layers parallel to ‘bioclimatic’ variables (i.e. mean, maximum, minimum, range, and standard deviation) over the entire period, plus a parallel data set (mean, maximum, minimum, range, standard deviation) over spring 2013 (March-May) only, the period which we believe most relevant to the H7N9 outbreak.

### Ecological niche modeling

We used a maximum entropy algorithm (Maxent; [14]) to estimate an ecological ‘niche’ for H7N9 virus. Maxent follows the principle of maximum entropy, spreading out probabilities as uniformly as possible, subjects to the constraints of observed values (i.e. known occurrences). We thresholded model predictions to produce binary maps by assessing the highest suitability value at which 90% of input occurrence points were included in the predicted suitable area (i.e. Maxent's 10% training presence threshold). This approach is conservative in estimating ecological niches, as it eliminates extreme values that may result from erroneous identifications or georeferencing [15].

To evaluate niche models, we used a spatial subsetting approach to erect a rigorous test of model predictions: we sorted occurrences by latitude and separately by longitude, and set aside the central third as evaluation data, using the remaining two-thirds for model calibration. Hence, we developed two evaluation data sets (i.e. subsetting by latitude and subsetting by longitude) for models produced in relation to the two NDVI datasets, for a total of four tests. Significance was assessed by means of a cumulative binomial test, comparing success in anticipating the spatial distribution of known occurrences against null expectations based on the proportional area identified as suitable. A final niche model was calibrated using all of the 77 records.

## Results

Most H7N9 cases have been reported from Shanghai, Zhejiang, and Jiangsu provinces, from where occurrences were aggregated, whereas occurrences in Beijing, Shandong, Henan, Hunan, Jiangxi, and Fujian provinces were more sporadic and scattered (Figure 1). Environmental variation across the range of H7N9 is quite dramatic (Figure 1), with highest NDVI values observed in southern China; the independent variation in the principal components can be appreciated in Figure 1.

**Figure 2:**
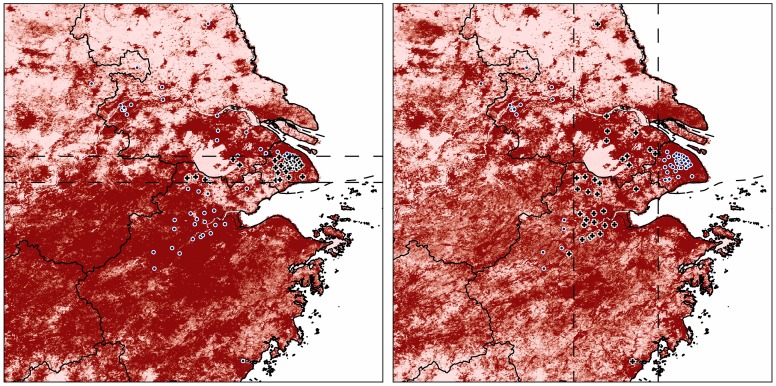
Model performance in anticipating the geographic distribution of independent latitude (left) and longitude (right) records using the principal component axes. Blue dots were sites used to calibrate the niche model, black crosses indicate independent testing records (inner panels), dark red suggests high suitability.

In model evaluations, our niche models showed good performance in anticipating the spatial distribution of independent testing records in three of the four tests (*P*<0.05; Table 1; Figure 2). Omission rates in models with longitude-based subsetting were higher than in models with latitude-based subsetting (Table 1), which might result from the broader geographic spread in longitude (Figure 2). In final model predictions, environmental variables with highest gain [14] in isolation were principal component 5 and standard deviation of the entire period; environmental variables that decreased gain most when omitted were principal component 1 and standard deviation of the entire period.

**Table 1:** Model performance in anticipating a ‘left out’ third of distributions of cases of H7N9 with respect to latitude and longitude.

Subsetting criterion	Layer set	10% training presence threshold*	Omission on evaluation data	*P*
**Latitude**	PCA	24.656	6/32	<0.05
**Latitude**	bioNDVI	13.998	6/32	<0.05
**Longitude**	PCA	19.56	11/32	<0.1
**Longitude**	bioNDVI	14.016	10/32	<0.05

*Maxent cumulative output.

**Figure 3:**
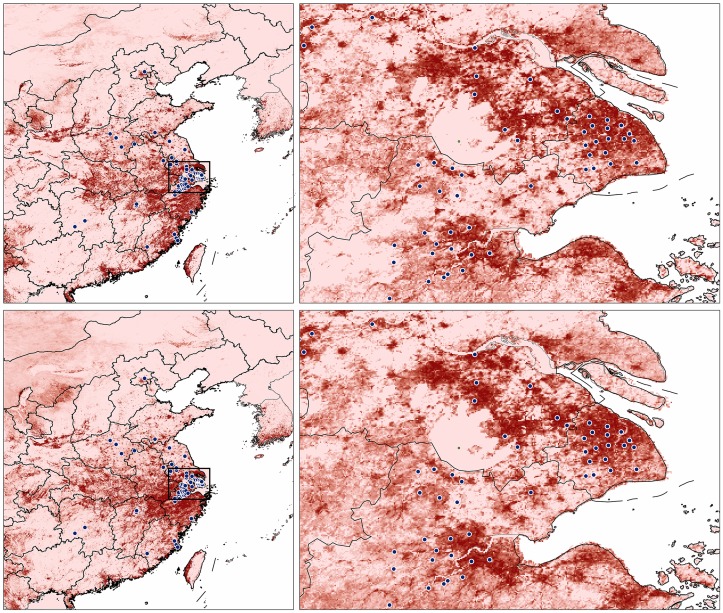
Final suitability model predictions for H7N9 across eastern China using principal components (top row) and the bioNDVI dataset (bottom row). Right-hand panels show greater detail for the areas indicated in the left-hand panels; dark red indicates zones of high suitability.

In final models, models based on both environmental datasets identified a particularly suitable zone in east-central China, including Shanghai, Zhejiang, southern Jiangsu, northwestern Fujian and Jiangxi, and parts of Anhui provinces (Figure 3). The model based on the ‘bioNDVI’ dataset was somewhat more conservative in predictions. Hubei Province was identified as highly suitable, although no H7N9 cases have been reported there (Figure 3). Suitable areas in the north were distributed less continuously, whereas suitable areas in the south were only along the coast. Western Taiwan was also identified as highly suitable by models based on both environmental datasets (Figure 3).

**Figure 4:**
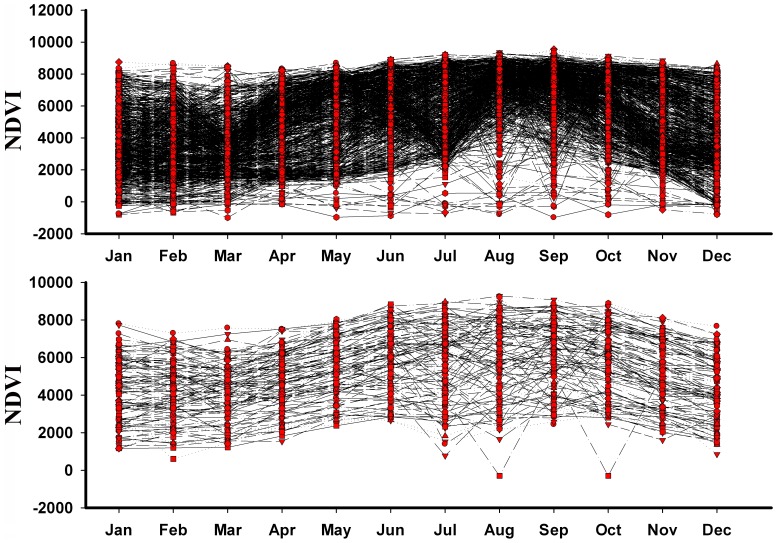
Visualization of year-round trends in MODIS NDVI greenness indices in modeled unsuitable (top) and suitable (bottom) areas for H7N9 virus based on a 10% omission threshold.

Attempting to visualize conditions under which models identified suitability, suitable areas tended to present conditions that were generally constant through the year (i.e. not much seasonal variation), with only gentle seasonal fluctuations. Unsuitable areas, in contrast, showed much more seasonal variation: NDVI values rose sharply in June–August and declined during September–December (Figure 4).

## Discussion

Limitations on the materials employed in this study have to be addressed here. The general distributional pattern of H7N9 has not been understood in detail to date, such that the distributional pattern so far has manifested sporadic and small clusters (Figure 1). Knowledge about the main virus reservoirs and the extent and distribution of the virus in animals remains limited, in spite of considerable efforts (e.g. [8]), it is likely that most human cases were exposed to the H7N9 virus through contact with infected poultry or contaminated environments, including markets that sell live poultry [16]. However clear links between infections in poultry and human cases have been difficult to establish, because this virus does not appear to cause clinical signs in infected poultry. Information to date also does not support sustained human-to-human transmission [17]. Effective and predictive risk maps can provide a useful means by which to design targeted surveillance efforts [3]–[6], and ecological niche modeling approaches are offering novel views of the geography of potential for disease transmission [9], [18]. Many aspects of the natural history, geography, and ecology of H7N9 virus remain poorly known or unknown; in such cases, niche modeling can only be applied to the outputs of the overall cycle as a “black box”, attempting to capture the integration of relevant niche components in H7N9 virus. Consisting with the regression model, which integrated agro-ecological, environmental and meteorological factors to map the spread potential of H7N9 [5]–[6], our niche model also identified the central coastal areas in Guangdong Province as high suitable, which were validated by the new emerging cases in 2014.

The First Law of Geography was introduced by Tobler [19], stating that everything is related to everything else, but that near things are more related than distant things; recent extension was to add dimensions of time in understanding and expressing distance and adjacency, resulting in a concept of spatiotemporal proximity proposed during the SARS (Severe Acute Respiratory Syndrome) outbreaks [20]. Here, we developed multitemporal vegetation index datasets to match the H7N9 outbreaks in spatiotemporal coverage; the hope was to characterize ecological requirements of H7N9 optimally, although many questions concerning the sporadic outbreaks of H7N9 remain up in the air. Using these datasets, models showed good performance in anticipating the known geographic distribution of independent occurrence records (including the new emerging cases in January 2014); however, given that H7N9 is likely still in the process of spreading out to its full distributional and environmental potential, we are most confident in our result as near-term and local solution rather than as global summaries.

For H5N1, researchers integrated large data sets combining information on poultry trade routes, numbers of birds being transported, distribution of live-bird markets, supply routes, waterfowl numbers, land use, and human population densities to trace H5N1 transmission (e.g. [21]–[22]). For H7N9, such analyses are only just beginning [3]–[6], particularly as sources of data on the virus and routes by which it infects humans remain unclear. Birds at live poultry markets have been suspected as a source, but broad testing of poultry and other animals have not turned up significant infection rates [3]; only isolates from Shanghai live poultry markets showed high homology with viruses causing human infections [8]. Based on experience with H5N1, Butler [3] suggested that high suitability areas for H7N9 might include Shandong Province and a belt extending around the Bohai Sea to Liaoning Province in the north.

However, H5N1 dynamics offer only a starting point for identifying areas at risk, since ecological requirements and natural history of H5N1 and H7N9 might well be different. Our models identified high-risk zones for H7N9 in east-central China. Continued vigilance therefore is needed within affected and neighboring areas to detect infections in animals and humans, as the current distributional pattern suggest that H7N9 would be in previously affected and possibly neighboring areas, and potentially in travelers from these areas returning to other countries. Attention should be paid to transmission of H7N9 between the mainland and Taiwan, since highly suitable areas were observed along both the eastern seaboard of mainland China and in western Taiwan.

## Supporting Information

Table S1 **H7N9 case occurrences used in ecological niche modeling.**
(DOC)
