# Supplementary material for: Potential Geographic Distribution of the Novel Avian-Origin Influenza A (H7N9) Virus
Source: PLoS One. 2014 Apr 1;9(4):e93390. doi: 10.1371/journal.pone.0093390 (PMC3972139; doi:10.1371/journal.pone.0093390)
Supplement: Table S1 — H7N9 case occurrences used in ecological niche modeling. (DOC) [file pone.0093390.s001.doc]

**Table S1 H7N9 case occurrences used in ecological niche modeling.**

| **Province** | **Locality** | **Latitude** | **Longitude** | **Source** |
| --- | --- | --- | --- | --- |
| Zhejiang | Wenzhou | 27.994267 | 120.699367 | <http://goo.gl/maps/ZsVW8> |
| Zhejiang | Jiande, Hangzhou | 29.474765 | 119.281212 | [http://goo.gl/maps/ZsVW9](http://goo.gl/maps/ZsVW8) |
| Zhejiang | Hangzhou | 29.716681 | 119.685059 | [http://goo.gl/maps/ZsVW10](http://goo.gl/maps/ZsVW8) |
| Zhejiang | Hangzhou | 29.735762 | 119.267578 | [http://goo.gl/maps/ZsVW11](http://goo.gl/maps/ZsVW8) |
| Zhejiang | Hangzhou | 29.802518 | 119.564209 | [http://goo.gl/maps/ZsVW12](http://goo.gl/maps/ZsVW8) |
| Zhejiang | Hangzhou | 29.945415 | 119.893799 | [http://goo.gl/maps/ZsVW13](http://goo.gl/maps/ZsVW8) |
| Zhejiang | Hangzhou | 29.981108 | 120.022888 | [http://goo.gl/maps/ZsVW14](http://goo.gl/maps/ZsVW8) |
| Zhejiang | Hangzhou | 30.007274 | 120.055847 | [http://goo.gl/maps/ZsVW15](http://goo.gl/maps/ZsVW8) |
| Zhejiang | Hangzhou | 30.038189 | 120.18219 | [http://goo.gl/maps/ZsVW16](http://goo.gl/maps/ZsVW8) |
| Zhejiang | Hangzhou | 30.107118 | 119.599915 | [http://goo.gl/maps/ZsVW17](http://goo.gl/maps/ZsVW8) |
| Zhejiang | Hangzhou | 30.17125 | 120.256348 | [http://goo.gl/maps/ZsVW18](http://goo.gl/maps/ZsVW8) |
| Zhejiang | Hangzhou | 30.183122 | 119.926758 | [http://goo.gl/maps/ZsVW19](http://goo.gl/maps/ZsVW8) |
| Zhejiang | Hangzhou | 30.185496 | 120.410156 | [http://goo.gl/maps/ZsVW20](http://goo.gl/maps/ZsVW8) |
| Zhejiang | Hangzhou | 30.221102 | 120.08606 | [http://goo.gl/maps/ZsVW21](http://goo.gl/maps/ZsVW8) |
| Zhejiang | Hangzhou | 30.25195 | 119.605408 | [http://goo.gl/maps/ZsVW22](http://goo.gl/maps/ZsVW8) |
| Zhejiang | Hangzhou | 30.262626 | 119.959717 | [http://goo.gl/maps/ZsVW23](http://goo.gl/maps/ZsVW8) |
| Zhejiang | Hangzhou | 30.356286 | 119.86084 | [http://goo.gl/maps/ZsVW24](http://goo.gl/maps/ZsVW8) |
| Zhejiang | Hangzhou | 30.363396 | 120.080566 | [http://goo.gl/maps/ZsVW25](http://goo.gl/maps/ZsVW8) |
| Zhejiang | Hangzhou | 30.401307 | 120.237122 | [http://goo.gl/maps/ZsVW26](http://goo.gl/maps/ZsVW8) |
| Zhejiang | Huzhou | 30.670991 | 120.135498 | [http://goo.gl/maps/ZsVW27](http://goo.gl/maps/ZsVW8) |
| Zhejiang | Huzhou | 30.708781 | 119.987183 | [http://goo.gl/maps/ZsVW28](http://goo.gl/maps/ZsVW8) |
| Zhejiang | Huzhou | 30.751278 | 119.819641 | [http://goo.gl/maps/ZsVW29](http://goo.gl/maps/ZsVW8) |
| Zhejiang | Jiaxing | 30.753924 | 120.758543 | [http://goo.gl/maps/ZsVW30](http://goo.gl/maps/ZsVW8) |
| Zhejiang | Huzhou | 30.845647 | 120.17395 | [http://goo.gl/maps/ZsVW31](http://goo.gl/maps/ZsVW8) |
| Shanghai | Shanghai | 30.892797 | 121.223145 | [http://goo.gl/maps/ZsVW32](http://goo.gl/maps/ZsVW8) |
| Shanghai | Shanghai | 30.902225 | 121.289063 | [http://goo.gl/maps/ZsVW33](http://goo.gl/maps/ZsVW8) |
| Zhejiang | Huzhou | 30.903035 | 120.094299 | [http://goo.gl/maps/ZsVW34](http://goo.gl/maps/ZsVW8) |
| Zhejiang | Huzhou | 30.930501 | 119.805908 | [http://goo.gl/maps/ZsVW35](http://goo.gl/maps/ZsVW8) |
| Shanghai | Shanghai | 30.939924 | 121.431885 | [http://goo.gl/maps/ZsVW36](http://goo.gl/maps/ZsVW8) |
| Shanghai | Shanghai | 30.949347 | 121.651611 | [http://goo.gl/maps/ZsVW37](http://goo.gl/maps/ZsVW8) |
| Zhejiang | Huzhou | 30.949347 | 119.940491 | [http://goo.gl/maps/ZsVW38](http://goo.gl/maps/ZsVW8) |
| Shanghai | Shanghai | 31.015279 | 121.256104 | [http://goo.gl/maps/ZsVW39](http://goo.gl/maps/ZsVW8) |
| Shanghai | Shanghai | 31.034108 | 121.387939 | [http://goo.gl/maps/ZsVW40](http://goo.gl/maps/ZsVW8) |
| Shanghai | Shanghai | 31.083518 | 121.555481 | [http://goo.gl/maps/ZsVW41](http://goo.gl/maps/ZsVW8) |
| Shanghai | Shanghai | 31.08587 | 121.478577 | [http://goo.gl/maps/ZsVW42](http://goo.gl/maps/ZsVW8) |
| Shanghai | Shanghai | 31.088222 | 121.445618 | [http://goo.gl/maps/ZsVW43](http://goo.gl/maps/ZsVW8) |
| Shanghai | Shanghai | 31.09763 | 121.220398 | [http://goo.gl/maps/ZsVW44](http://goo.gl/maps/ZsVW8) |
| Shanghai | Shanghai | 31.099982 | 121.343994 | [http://goo.gl/maps/ZsVW45](http://goo.gl/maps/ZsVW8) |
| Shanghai | Shanghai | 31.116443 | 121.59668 | [http://goo.gl/maps/ZsVW46](http://goo.gl/maps/ZsVW8) |
| Shanghai | Shanghai | 31.128199 | 121.308289 | [http://goo.gl/maps/ZsVW47](http://goo.gl/maps/ZsVW8) |
| Shanghai | Shanghai | 31.135252 | 121.629639 | [http://goo.gl/maps/ZsVW48](http://goo.gl/maps/ZsVW8) |
| Jiangsu | Suzhou | 31.137729 | 120.645222 | [http://goo.gl/maps/ZsVW49](http://goo.gl/maps/ZsVW8) |
| Shanghai | Shanghai | 31.151707 | 121.547241 | [http://goo.gl/maps/ZsVW50](http://goo.gl/maps/ZsVW8) |
| Shanghai | Shanghai | 31.151707 | 121.413345 | [http://goo.gl/maps/ZsVW51](http://goo.gl/maps/ZsVW8) |
| Shanghai | Shanghai | 31.166985 | 121.485443 | [http://goo.gl/maps/ZsVW52](http://goo.gl/maps/ZsVW8) |
| Shanghai | Shanghai | 31.192833 | 121.397552 | [http://goo.gl/maps/ZsVW53](http://goo.gl/maps/ZsVW8) |
| Shanghai | Shanghai | 31.194008 | 121.591187 | [http://goo.gl/maps/ZsVW54](http://goo.gl/maps/ZsVW8) |
| Shanghai | Shanghai | 31.198706 | 121.470337 | [http://goo.gl/maps/ZsVW55](http://goo.gl/maps/ZsVW8) |
| Shanghai | Shanghai | 31.208691 | 121.523895 | [http://goo.gl/maps/ZsVW56](http://goo.gl/maps/ZsVW8) |
| Shanghai | Shanghai | 31.210452 | 121.209412 | [http://goo.gl/maps/ZsVW57](http://goo.gl/maps/ZsVW8) |
| Jiangsu | Suzhou | 31.229243 | 120.539246 | [http://goo.gl/maps/ZsVW58](http://goo.gl/maps/ZsVW8) |
| Shanghai | Shanghai | 31.240398 | 121.562347 | [http://goo.gl/maps/ZsVW59](http://goo.gl/maps/ZsVW8) |
| Shanghai | Shanghai | 31.240985 | 121.405106 | [http://goo.gl/maps/ZsVW60](http://goo.gl/maps/ZsVW8) |
| Shanghai | Shanghai | 31.25977 | 121.441498 | [http://goo.gl/maps/ZsVW61](http://goo.gl/maps/ZsVW8) |
| Shanghai | Shanghai | 31.262118 | 121.278076 | [http://goo.gl/maps/ZsVW62](http://goo.gl/maps/ZsVW8) |
| Shanghai | Shanghai | 31.265639 | 121.521835 | [http://goo.gl/maps/ZsVW63](http://goo.gl/maps/ZsVW8) |
| Shanghai | Shanghai | 31.278551 | 121.365967 | [http://goo.gl/maps/ZsVW64](http://goo.gl/maps/ZsVW8) |
| Jiangsu | Suzhou | 31.280898 | 120.613403 | [http://goo.gl/maps/ZsVW65](http://goo.gl/maps/ZsVW8) |
| Shanghai | Shanghai | 31.283245 | 121.49231 | [http://goo.gl/maps/ZsVW66](http://goo.gl/maps/ZsVW8) |
| Shanghai | Shanghai | 31.297328 | 121.445618 | [http://goo.gl/maps/ZsVW67](http://goo.gl/maps/ZsVW8) |
| Shanghai | Shanghai | 31.311408 | 121.404419 | [http://goo.gl/maps/ZsVW68](http://goo.gl/maps/ZsVW8) |
| Jiangsu | Kunshan, Suzhou | 31.320794 | 121.063843 | [http://goo.gl/maps/ZsVW69](http://goo.gl/maps/ZsVW8) |
| Shanghai | Shanghai | 31.341909 | 121.253357 | [http://goo.gl/maps/ZsVW70](http://goo.gl/maps/ZsVW8) |
| Jiangsu | Kunshan, Suzhou | 31.384913 | 120.98115 | [http://goo.gl/maps/ZsVW71](http://goo.gl/maps/ZsVW8) |
| Jiangsu | Binhu, Wuxi | 31.527954 | 120.282692 | [http://goo.gl/maps/ZsVW72](http://goo.gl/maps/ZsVW8) |
| Jiangsu | Changshu, Suzhou | 31.653584 | 120.752507 | [http://goo.gl/maps/ZsVW73](http://goo.gl/maps/ZsVW8) |
| Jiangsu | Huishan, Wuxi | 31.681019 | 120.298487 | [http://goo.gl/maps/ZsVW74](http://goo.gl/maps/ZsVW8) |
| Jiangsu | Jiangyin, Wuxi | 31.920673 | 120.284946 | [http://goo.gl/maps/ZsVW75](http://goo.gl/maps/ZsVW8) |
| Jiangsu | Jiangning, Nanjing | 31.940509 | 118.840485 | [http://goo.gl/maps/ZsVW76](http://goo.gl/maps/ZsVW8) |
| Jiangsu | Qinhuai, Nanjing | 32.011811 | 118.798656 | [http://goo.gl/maps/ZsVW77](http://goo.gl/maps/ZsVW8) |
| Jiangsu | Qinhuai, Nanjing | 32.01414 | 118.763637 | [http://goo.gl/maps/ZsVW78](http://goo.gl/maps/ZsVW8) |
| Jiangsu | Qinhuai, Nanjing | 32.023455 | 118.806896 | [http://goo.gl/maps/ZsVW79](http://goo.gl/maps/ZsVW8) |
| Jiangsu | Baixia, Nanjing | 32.04169 | 118.796253 | [http://goo.gl/maps/ZsVW80](http://goo.gl/maps/ZsVW8) |
| Jiangsu | Xuanwu, Nanjing | 32.048629 | 118.797859 | [http://goo.gl/maps/ZsVW81](http://goo.gl/maps/ZsVW8) |
| Jiangsu | Xiaguan, Nanjing | 32.088279 | 118.739572 | [http://goo.gl/maps/ZsVW82](http://goo.gl/maps/ZsVW8) |
| Jiangsu | Qixian, Nanjing | 32.096168 | 118.908493 | [http://goo.gl/maps/ZsVW83](http://goo.gl/maps/ZsVW8) |
| Jiangsu | Runzhou, Zhenjiang | 32.176737 | 119.417438 | [http://goo.gl/maps/ZsVW84](http://goo.gl/maps/ZsVW8) |
| Jiangsu | Yangzhou | 32.377974 | 119.404726 | [http://goo.gl/maps/ZsVW85](http://goo.gl/maps/ZsVW8) |
| Anhui | Nanqiao, Chuzhou | 32.434048 | 118.275171 | [http://goo.gl/maps/ZsVW86](http://goo.gl/maps/ZsVW8) |
| Anhui | Tianchang, Chuzhou | 32.689438 | 119.003686 | [http://goo.gl/maps/ZsVW87](http://goo.gl/maps/ZsVW8) |
| Jiangsu | Tinghu, Yancheng | 33.391508 | 120.13994 | [http://goo.gl/maps/ZsVW88](http://goo.gl/maps/ZsVW8) |
| Henan | Zhoukou | 33.618686 | 114.657616 | [http://goo.gl/maps/ZsVW89](http://goo.gl/maps/ZsVW8) |
| Anhui | Qiaocheng,Bozhou | 33.876235 | 115.779025 | [http://goo.gl/maps/ZsVW90](http://goo.gl/maps/ZsVW8) |
| Jiangsu | Shuyang, Suqiang | 34.111022 | 118.804784 | [http://goo.gl/maps/ZsVW91](http://goo.gl/maps/ZsVW8) |
| Henan | Weishi, Kaifeng | 34.411494 | 114.193081 | [http://goo.gl/maps/ZsVW92](http://goo.gl/maps/ZsVW8) |
| Henan | Guancheng, Zhengzhou | 34.7545 | 113.677548 | [http://goo.gl/maps/ZsVW93](http://goo.gl/maps/ZsVW8) |
| Beijing | Shunyi | 40.130211 | 116.654812 | [http://goo.gl/maps/ZsVW94](http://goo.gl/maps/ZsVW8) |
| Fujian | Longyan | 24.974058 | 116.877006 | Internet and literature |
| Fujian | Fuqing, Shanshan | 25.529302 | 119.521377 | Internet and literature |
| Fujian | Fuqing, Yangxia | 25.736873 | 119.390879 | Internet and literature |
| Fujian | Changshan, Jianxin | 26.067117 | 119.238098 | Internet and literature |
| Hunan | Shaoyang, Wugang | 26.726599 | 110.631884 | Internet and literature |
| Hunan | Shaoyang, Shuangqing | 27.232708 | 111.496341 | Internet and literature |
| Jiangxi | Nanchangxian | 28.545459 | 115.944162 | Internet and literature |
| Jiangxi | Nanchang, Qingshanhu | 28.682985 | 115.962144 | Internet and literature |
| Shandong | Zaozhuang, Shizhongqu | 34.864114 | 117.556123 | Internet and literature |
